# Draft Genome Sequences of Two *Sphingobium* Species Associated with Hexachlorocyclohexane (HCH) Degradation Isolated from an HCH-Contaminated Soil

**DOI:** 10.1128/mra.00886-21

**Published:** 2022-02-17

**Authors:** Nelson Khan, Rodolfo Brizola Toscan, Accadius Lunayo, Benson Wamalwa, Edward Muge, Francis J. Mulaa, René Kallies, Hauke Harms, Lukas Y. Wick, Ulisses Nunes da Rocha

**Affiliations:** a Department of Biochemistry, University of Nairobi, Nairobi, Kenya; b Department of Chemistry, University of Nairobi, Nairobi, Kenya; c Center for Biotechnology and Bioinformatics, University of Nairobi, Nairobi, Kenya; d Department of Environmental Microbiology, Helmholtz Centre for Environmental Research—UFZ, Leipzig, Germany; Montana State University

## Abstract

The draft genome sequences of two *Sphingobium* strains that are hexachlorocyclohexane (HCH) degraders are presented. The strains were isolated from HCH-contaminated soil in Kitengela, Kenya. Both genomes possess the *lin* genes responsible for HCH degradation and gene clusters for degradation of other xenobiotic compounds.

## ANNOUNCEMENT

Bioremediation of biodegradable lindane (γ-hexachlorocyclohexane [HCH]) is a viable detoxification strategy for maintaining environmental health (1, 2). Several microorganisms can degrade HCH isomers (3, 4). *Sphingomonadaceae* seem to play a central role in the complete mineralization of HCH, with the catabolic genes initially identified in Sphingobium japonicum UT26 (5).

Here, two *Sphingobium* species (*Sphingobium* sp. strains S6 and S8) were isolated from HCH-contaminated soil collected from an obsolete former pesticide store in Kitengela, Kenya (01.49 S, 37.048 E). For bacterial isolation, we used a minimum salt medium (MSM) (6) spiked with 100 μg/mL γ-HCH. Pure colonies were obtained by spreading serial dilutions (10^−3^ to 10^−6^) of the enrichment cultures onto 1:10 diluted Luria-Bertani (LB) agar plates supplemented with 100 μg/mL γ-HCH, followed by incubation at 30°C for 72 h. The HCH degradation capacity was assessed by dechlorinase assay according to the method of Phillips et al. (7) and in the liquid medium following Boltner et al. (8).

Genomic DNA was extracted from 48-h LB cultures grown at 30°C using a Wizard genomic DNA purification kit (Promega, USA). DNA was quantified using a Qubit fluorometer (Thermo Fisher Scientific, USA). According to the manufacturer’s instructions, a NEBNext Ultra II FS DNA library kit (New England Biolabs, USA) was used to prepare a paired-end 300-bp library for genome sequencing on an Illumina MiSeq platform. We used Sickle v1.33 (9) with a Phred quality score of >30 for sequence trimming. *De novo* sequence assembly was performed using SPAdes v3.15.2 (10), while CheckM v1.0.18 and RefineM v0.0.25 (11) were used for quality checking and to provide completeness and contamination information, respectively. The genomes were annotated using PROKKA v1.14.5 (12) and the Rapid Annotation using Subsystems Technology toolkit (RASTtk) v2.0 (13). Unless otherwise stated, default parameter settings were applied for all software used.

The draft genomes of *Sphingobium* sp. strains S6 and S8 had 42 and 44 contigs, with total lengths of 4,173,956 bp and 4,170,555 bp, respectively. Both genomes showed 99.2% completeness and 2.06% contamination. The GC contents of *Sphingobium* sp. strains S6 and S8 were 62.4% and 62.53%, respectively. The annotations are summarized in Table 1. Previous reports show that HCH-degrading sphingomonads share the same degradation pathway that requires genes *linA* through *linF* (3, 8). Analysis of the draft genomes of the *Sphingobium* species described in this study revealed the presence of one copy each of *linA*, *linB*, *linC*, *linD*, *linE*, and *linF*, and two copies each of *linG*, *linH*, *linJ*, and *linX* per genome. Annotation using RASTtk showed gene clusters for the potential degradation of xenobiotic compounds such as 1,1,1-trichloro-2,2-bis(4-chlorophenyl) ethane (DDT); 1,4-dichlorobenzene; tetrachloroethene; 2,4-dichlorobenzoate; fluorobenzoate; benzoate; toluene; and xylene. In addition, the genetic potential for the production of carotenoids was predicted using antiSMASH v6.0.0 (14) in both strains.

**TABLE 1:** Summary of the draft whole-genome sequences of HCH-degrading *Sphingobium* strains isolated from an HCH-contaminated site in Kitengela, Kenya

Strain	GenBank accession no.	No. of contigs	*N*_50_ (bp)	Avg coverage (×)	Genome size (bp)	%GC	No. of rRNAs	No. of tRNAs	No. of tmRNAs^*a*^	No. of coding sequences	Transposon families	Closest phylogenetic neighbors (%ANI)^*b*^
*Sphingobium* sp. strain S6	CAJHOG000000000	44	538,820	48.72	4,173,956	62.40	3	49	1	4,015	IS*21* (*n* = 2), ISNCY (*n* = 1), IS*3* (*n* = 2), IS*5* (*n* = 1), IS*630* (*n* = 1), IS*256* (*n* = 1), IS*481* (*n* = 1)	*Sphingobium* sp. strain S8 (99.96), Sphingobium indicum B90A (85.93) (16), Sphingobium japonicum UT26S (85.51) (17), Sphingobium quisquiliarum P25 (83.88) (18), Sphingobium chlorophenolicum L-1 (83.65) (19)
*Sphingobium* sp. strain S8	CAJHOH000000000	48	454,689	36.73	4,170,555	62.53	3	47	1	4,039	IS*21* (*n* = 2), IS*5* (*n* = 1), IS*630* (*n* = 1), IS*256* (*n* = 1), IS*481* (*n* = 1)	*Sphingobium* sp. strain S6 (99.96), Sphingobium indicum B90A (85.76) (16), Sphingobium japonicum UT26S (85.41) (17), Sphingobium quisquiliarum P25 (83.85) (18), Sphingobium chlorophenolicum L-1 (83.57) (19)

a tmRNAs, transfer-messenger RNAs.

b Phylogenetically related neighbors were computed based on average nucleotide identity (ANI) [20] analysis.

The availability of the genome sequences of the two *Sphingobium* species may be instrumental in promoting HCH degradation by mixed (multidomain) microbial communities such as fungal bacterial associations (15).

### Data availability.

These whole-genome shotgun projects have been deposited at ENA/DDBJ/GenBank under accession numbers CAJHOG000000000 and CAJHOH000000000 for *Sphingobium* sp. strains S6 and S8, respectively. The versions described in this paper are CAJHOG000000000.1 and CAJHOH000000000.1 for *Sphingobium* sp. strains S6 and S8, respectively. The raw data are available at ENA under SRA accession numbers ERR4392070 and ERR4392071. All project data are available under BioProject accession number PRJEB39494.
